# Cancer of Pylorus—Obscurity of Symptoms—Suppuration of Gall-bladder

**Published:** 1847-01

**Authors:** 


*Cancer of Pylorus—Obscurity of Symptoms—Suppuration of Gall-bladder.*—Dr. Francis sent for exhibition a fine specimen of cancerous ulcer which involved the whole of the pylorus. Its surface was ragged and burrowing, the margins raised and everted; in length it measured four inches, in breadth two. The stomach was pale, and unusually small; the duodenum was of a deep slate colour.

It was taken from the body of a man, æt. 60, who, within the last eighteen months, had suffered much from privation and severe domestic troubles. He was under the daily and close observation of Dr. Francis for the last six months of his life, during which time he gradually emaciated. There was never any vomiting or complaint of pain, or other symptom indicative of local disease, but he was throughout the subject of a settled gloom, under the influence of which he seemed to be slowly sinking. A few weeks before death he contracted diarrhœa, which was then epidemic.

Dr. Francis considers that a very interesting and instructive feature in this case was the absence of local manifestations of disease, notwithstanding the existence of so formidable a lesion in a part of the stomach so almost uniformly attended with vomiting or pain. It added another to many instances he had had the opportunity of observing at the workhouse, of the fact, that where strong grief had taken possession of the mind it sometimes happened that sensibility seemed to have become so impaired that the symptoms ordinarily resulting from local irritation had not been developed. In several inspections of persons who had died whilst labouring under the melancholic form of insanity, or under that particular state of mind which, in popular language, is described as "a broken heart," he had never failed to find organic disease of some part or other, however obscure its existence might have been during life.

Along with the cancer of the stomach, the gall-bladder was found to be distended with a puriform fluid untinged with bile, which, under the microscope, was seen to contain pus, mucus, and inflammation globules, and behaved as pus on the addition of liq. potassæ. Several superficial granulating ulcers were observed upon the inner surface of the viscus, and, a short distance within its duct, was firmly impacted a round calculus, nearly a third of an inch in diameter, and composed chiefly of cholesterine. The liver did not seem to be unhealthy, and there was no cancer elsewhere than in the stomach.

*Ibid*

